# Mitsui-7, heat-treated, and nitrogen-doped multi-walled carbon nanotubes elicit genotoxicity in human lung epithelial cells

**DOI:** 10.1186/s12989-019-0318-0

**Published:** 2019-10-07

**Authors:** Katelyn J. Siegrist, Steven H. Reynolds, Dale W. Porter, Robert R. Mercer, Alison K. Bauer, David Lowry, Lorenzo Cena, Todd A. Stueckle, Michael L. Kashon, John Wiley, Jeffrey L. Salisbury, John Mastovich, Kristin Bunker, Mark Sparrow, Jason S. Lupoi, Aleksandr B. Stefaniak, Michael J. Keane, Shuji Tsuruoka, Mauricio Terrones, Michael McCawley, Linda M. Sargent

**Affiliations:** 1 Health Effects Laboratory Division, National Institute for Occupational Safety and Health, 1095 Willowdale Rd, Morgantown, WV 26505 USA; 2 Department of Occupational and Environmental Health Sciences, West Virginia University, Morgantown, WV 26506 USA; 3 Anschutz Medical Campus, Department of Environmental and Occupational Health, University of Colorado, Aurora, CO 80045 USA; 4 Department of Health, West Chester University, West Chester, PA 19383 USA; 5 Department of Pediatrics, East Carolina University, Greenville, NC 27834 USA; 6 Mayo Clinic, Rochester, MN 55905 USA; 7 Bruker AXS Inc, Madison, WI 53711 USA; 8 RJ Lee Group, 350 Hochberg Road, Monroeville, PA 15146 USA; 9 Independent Consultant, Allison Park, PA 15101 USA; 10 Respiratory Health Division, National Institute for Occupational Safety and Health, Morgantown, WV 26505 USA; 11 Shinshu University, Nagano City, Japan; 12 Pennsylvania State University, State College, PA 16801 USA

**Keywords:** Carbon nanotubes, Genotoxicity, Chromosomal translocations, Centromere, Aneuploidy, In vitro, Mitotic spindle, Cell cycle

## Abstract

**Background:**

The unique physicochemical properties of multi-walled carbon nanotubes (MWCNT) have led to many industrial applications. Due to their low density and small size, MWCNT are easily aerosolized in the workplace making respiratory exposures likely in workers. The International Agency for Research on Cancer designated the pristine Mitsui-7 MWCNT (MWCNT-7) as a Group 2B carcinogen, but there was insufficient data to classify all other MWCNT. Previously, MWCNT exposed to high temperature (MWCNT-HT) or synthesized with nitrogen (MWCNT-ND) have been found to elicit attenuated toxicity; however, their genotoxic and carcinogenic potential are not known. Our aim was to measure the genotoxicity of MWCNT-7 compared to these two physicochemically-altered MWCNTs in human lung epithelial cells (BEAS-2B & SAEC).

**Results:**

Dose-dependent partitioning of individual nanotubes in the cell nuclei was observed for each MWCNT material and was greatest for MWCNT-7. Exposure to each MWCNT led to significantly increased mitotic aberrations with multi- and monopolar spindle morphologies and fragmented centrosomes. Quantitative analysis of the spindle pole demonstrated significantly increased centrosome fragmentation from 0.024–2.4 μg/mL of each MWCNT. Significant aneuploidy was measured in a dose-response from each MWCNT-7, HT, and ND; the highest dose of 24 μg/mL produced 67, 61, and 55%, respectively. Chromosome analysis demonstrated significantly increased centromere fragmentation and translocations from each MWCNT at each dose. Following 24 h of exposure to MWCNT-7, ND and/or HT in BEAS-2B a significant arrest in the G1/S phase in the cell cycle occurred, whereas the MWCNT-ND also induced a G2 arrest. Primary SAEC exposed for 24 h to each MWCNT elicited a significantly greater arrest in the G1 and G2 phases. However, SAEC arrested in the G1/S phase after 72 h of exposure. Lastly, a significant increase in clonal growth was observed one month after exposure to 0.024 μg/mL MWCNT-HT & ND.

**Conclusions:**

Although MWCNT-HT & ND cause a lower incidence of genotoxicity, all three MWCNTs cause the same type of mitotic and chromosomal disruptions. Chromosomal fragmentation and translocations have not been observed with other nanomaterials. Because in vitro genotoxicity is correlated with in vivo genotoxic response, these studies in primary human lung cells may predict the genotoxic potency in exposed human populations.

**Electronic supplementary material:**

The online version of this article (10.1186/s12989-019-0318-0) contains supplementary material, which is available to authorized users.

## Background

Multi-walled carbon nanotubes (MWCNT) have been used and studied extensively given their unique physicochemical properties such as high aspect ratio, rigidity, strength, and electrical conductance [1]. Therefore, they are widely used for industrial applications leading to potential occupational exposures. However, due to their light-weight and small size they are prone to aerosolization leading to inhalation and potential risk for adverse human health effects, specifically respiratory disease [2]. Recently, the International Agency for Research on Cancer (IARC) designated the Mitsui-7 MWCNT (MWCNT-7), an extensively-studied pristine MWCNT material, as a Group 2B carcinogen or “possibly carcinogenic to humans” citing multiple studies that indicate tumor growth in rodents and genotoxicity relevant to humans [3]. A complete toxicity profile that includes the mechanism of genotoxicity is needed in order to properly identify the risk to exposed workers and to extrapolate that risk to numerous other non-pristine MWCNT materials. Physicochemically-altered MWCNTs elicit variable effects in the lung relating to cellular uptake, biocompatibility, cytotoxicity, oxidative stress, pulmonary inflammation, and fibrosis indicating carcinogenic potential for these materials as well [4–24]. However, there is insufficient evidence to determine the carcinogenic risk to humans exposed to these materials and even less evidence relating to the genotoxic mechanism. Consequently, all other MWCNTs have been labeled as Group 3 carcinogens or “not classifiable as to their carcinogenicity to humans”. Therefore, an investigation of the genotoxic mechanisms of MWCNT-7 and physicochemically-altered MWCNT materials is needed in models relevant to human occupational exposure.

Extensive in vivo and in vitro genotoxicity data have been reported from exposure to pristine MWCNT. Formation of micronuclei was found in A549 cells [25], primary human peripheral lymphocytes [26, 27], and RAW 264.7 macrophages [28] after exposure to pristine MWCNT in culture, as well as lung epithelial cells isolated from rats exposed via intratracheal installation [29, 30]. In each study, micronuclei were caused by clastogenic and aneugenic events indicating a dynamic mechanism of genotoxicity. Significant DNA breakage was observed via Comet assay in mouse alveolar macrophages exposed to pristine MWCNT in culture [31], lung cells isolated from mice exposed via intratracheal instillation [25], and rats exposed via nose-only inhalation [32]. Chinese hamster lung cells exposed to MWCNT-7, specifically, had a significantly greater percentage of bi- and multinucleated cells, as well as polyploidy [33]. Chromosome breakage was observed in RAW 264.7 macrophages following exposure to pristine MWCNT (10–20 nm) [28]. These investigations indicate a potential for a physical interaction between MWCNT material and the cell division apparatus, DNA, and other nuclear structures.

Previous in vitro studies have found that single-walled carbon nanotubes bind to G-C rich and telomeric regions of the chromosomes resulting in conformational changes in the DNA structure [34, 35]. Additionally, acid-oxidized MWCNTs form function hybrids with α- and β-tubulin, components of the microtubules, implicating a potential for interference with the mitotic spindle function [36]. Our previous research has shown that the same MWCNT material directly interacted with the mitotic spindle apparatus in human bronchial epithelial cells which led to multi- and monopolar mitotic divisions and fragmented centrosomes [37, 38]. These aberrant mitotic events led to cell cycle arrest in S-phase and aneuploidy in primary human lung epithelial cells [37]. Disruption of the mitotic spindle and aneuploidy in cultured cells is strongly correlated with in vivo carcinogenesis [39–42].

Altering the physicochemical properties of pristine MWCNT material has been shown to mitigate toxicity. Our laboratory has previously shown that MWCNT material 15 nm in diameter produces greater cytotoxicity and genotoxicity, specifically mitotic spindle aberration, aneuploidy, and cell cycle disruption, than the more-narrow SWCNT material 1 nm in diameter [37, 43, 44]. Given that the microtubules of the mitotic spindle are of similar diameter to the MWCNT material, it is reasonable that the alteration of diameter can have significant influences on the genotoxicity and carcinogenicity [45]. Heating MWCNT-7 material over 2000 °C (MWCNT-HT) increases crystallinity and purity of the individual structures [46–49], two alterations that could reduce the bioavailability and reactivity of the material. Doping MWCNT with nitrogen, either by incorporating nitrogen into the lattice structure of the nanotube wall during synthesis or by the addition of a nitrogen-containing functional group (MWCNT-ND) [50, 51], can alter the electronic properties, strength, as well as increase the hydrophilicity of the raw material [50–55]. Indeed, acid-oxidized MWCNT-ND material was shown to be less acutely toxic in the lung than undoped acid-oxidized MWCNT material in CD1 mice exposed to 1, 2.5, and 5 mg/kg through intratracheal installation [5]. A comparison of MWCNT-7 and ND material in immortalized small airway epithelial cells found the ND material to be less bioactive leading to differences in proliferation, cytotoxicity, ROS production, cell cycle, and total phosphor-tyrosine- and phosphor-threonine-altered proteins [56]. However, a two-year study of Wistar rats exposed to various MWCNT-HT materials through intraperitoneal injection found each material to produce an increase in tumor incidence greater than the positive control, amosite asbestos [20]. It should be noted that the authors found aspect ratio and curvature of the MWCNT-HT materials to be important factors regarding potency with shorter and tangled MWCNT-HT materials being relatively less toxic.

However, the effects of these physicochemical alterations on overall genotoxicity and the mechanism of genotoxicity have not been investigated. Therefore, in the present study we investigated the cytotoxicity, partitioning of individual nanotubes in the cell nuclei, cell cycle disruption, mitotic spindle disruption, and aneuploidy of MWCNT-HT and ND compared to MWCNT-7. The techniques used allowed for the quantitative analysis of spindle pole integrity, centromere fragmentation and translocations, as well as clonal growth as measures of carcinogenic potential.

## Results

### Characterization

#### Length and diameter

**Fig. 1:**
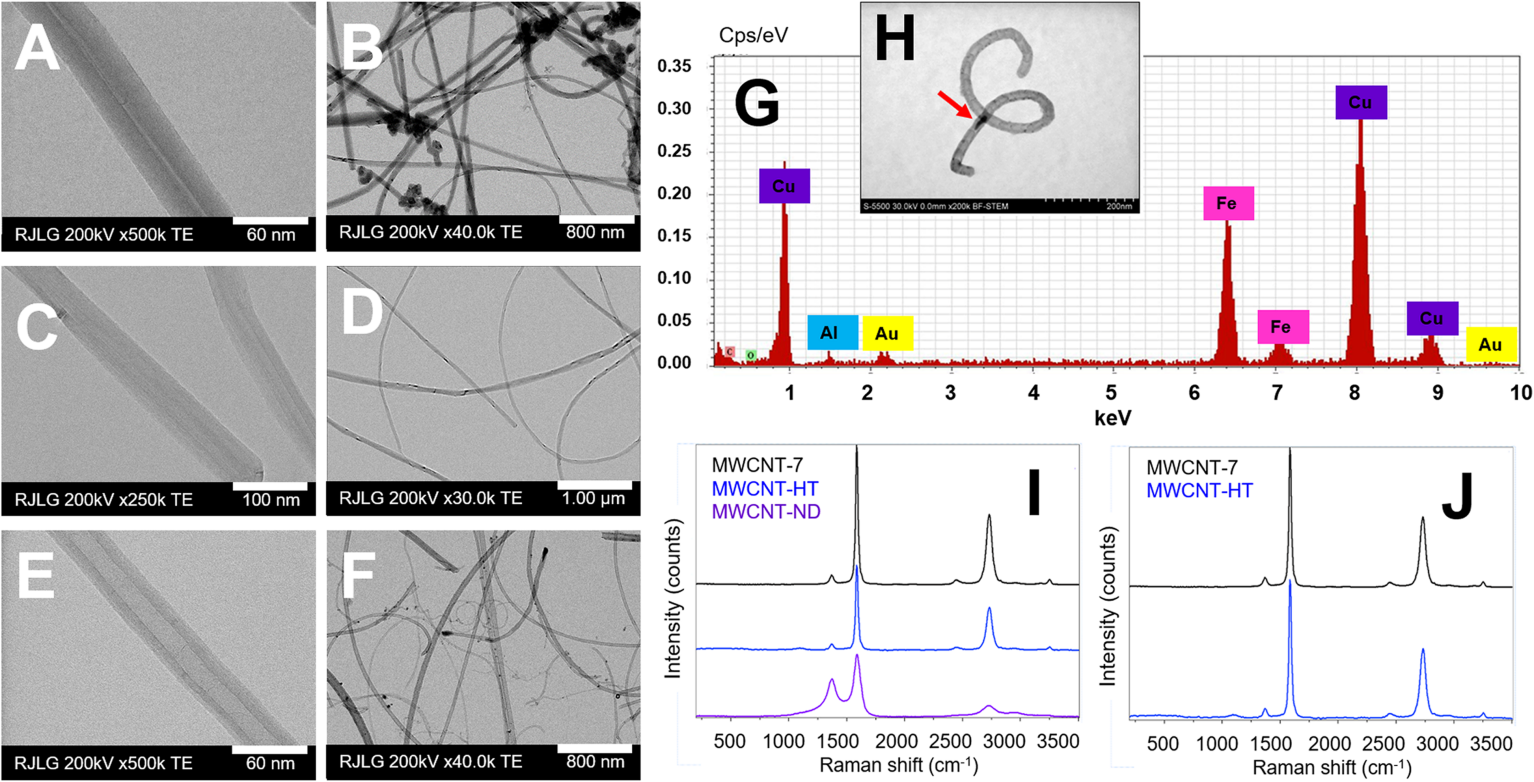
Physicochemical analysis of each MWCNT material. Electron micrographs of MWCNT-7 (**a** & **b**), HT (**c** & **d**), and ND (**e** & **f**). **g** EDS spectrum showing iron-rich catalyst contamination in the MWCNT-ND material. The copper in the spectrum is from the copper TEM grid. **h** DF-STEM image of a MWCNT-ND with red arrow pointing to iron-rich catalyst material. **i** & **j** Unique Raman spectra for each MWCNT material, D, G, and G’-bands. Magnification bar is 60 nm for **a**, **c**, & **e**. Magnification bar is 800 nm for **b**, **d**, & **f**

High-resolution scanning transmission electron microscopy (STEM) images showed a tubular structure with multiple walls for each MWCNT material (Fig. 1a-f). Diameter and length measurements of the MWCNT-7 were conducted previously [57]. The MWCNT-HT and -ND material were found to have similar physical dimensions (Table 1).

**Table 1:** Characterization of MWCNT Material

Measure		MWCNT-7	MWCNT-HT	MWCNT-ND
Mean diameter (nm)		49 ± 13^a^	57 ± 24	30 ± 23
Mean length (μm)		5 ± 4^a^	5 ± 4	2 ± 3
Hydrodynamic diameter (nm)		411 ± 16	499 ± 15	432 ± 18
Zeta potential (mV)		− 40 ± 1	− 51 ± 1	− 49 ± 2
Metal Contaminant (%Wt, SD)	Cr	0.023 ± 0.006	0.04 ± 0.01	0.020 ± 0.006
	Fe	0.020 ± 0.006	0.10 ± 0.03	0.09 ± 0.03
	Ni	0.020 ± 0.006	0.040 ± 0.006	0.08 ± 0.03
	Co	< 0.002 ± 0.000	< 0.002 ± 0.000	< 0.002 ± 0.000

MWCNT-7, HT, and ND mean diameter, mean length, hydrodynamic diameter, zeta potential, and metal catalyst contamination (ICP-MS) were measured; ± standard deviation, SD. ^a^Measured previously [57]

#### Purity

High-resolution STEM imaging identified residual catalyst material within the MWCNT-ND structure that was identified as iron through energy dispersive X-ray spectroscopy (EDS; Fig. 1g&h). Catalyst material was not observed by STEM imaging in the MWCNT-7 or HT samples (data not shown). ICP-MS data indicated that the MWCNT-7 material had 0.020% Cr, 0.020% Fe, and 0.020% Ni, the MWCNT-HT material had 0.040% Cr, 0.100% Fe, and 0.040% Ni, and the MWCNT-ND material had 0.020% Cr, 0.090% Fe, and 0.080% Ni (Table 1). Cobalt was not detected in any of the materials.

Raman spectroscopy determined unique spectra for each MWCNT material. The MWCNT-ND material demonstrated differences in the D, G, G’-band intensities compared to the MWCNT-7 and HT material. Figure 1i illustrates the differences in Raman band morphology of MWCNT-ND. The D-band, located near 1350 cm^− 1^ is more intense, and the G-band, near 1600 cm^− 1^ is wider. Additionally, the G’-peak near 2700 cm^− 1^ revealed a diminished intensity compared to the other MWCNT materials. These results are consistent with structural changes in the carbon, and specifically point to less graphitic, more amorphous carbon content. When normalized to the G-band, the spectra for MWCNT-7 and HT material were similar, however the peak intensity was lower in the MWCNT-HT material (Fig. 1i&j). These data indicate differences between the three MWCNT materials regarding the carbon structure. However, the D/G ratio does not change significantly for either material. Therefore, it is reasonable to conclude that the heat treatment and nitrogen doping does not significantly change the diameter of the carbon nanotubes.

#### Suspension properties

**Fig. 2:**
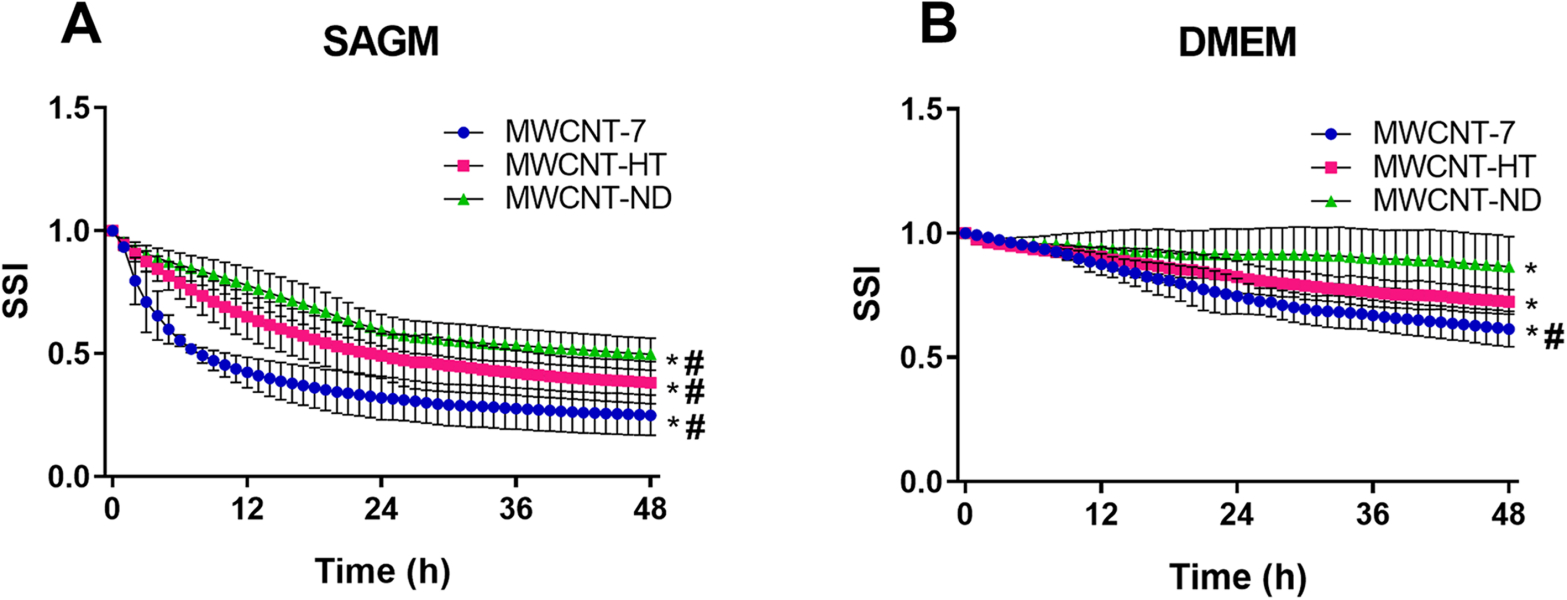
Suspension stability index of three MWCNT suspensions in two epithelial cell culture mediums. **a** All three MWCNT displayed significantly different stability curve parameter in SAGM (*p* ≤ 0.05). **b** MWCNT-7 displayed significantly less stability (p ≤ 0.05) than –HT and ND in DMEM. MWCNT-HT and – ND showed equivalent parallelism. Values were background corrected at each time point in a blank medium with vehicle control. * and # indicate curves with significantly different curve parameters and parallelism, respectively (p ≤ 0.05)

Dynamic light scattering analysis indicated suspension characteristics that varied for each of the three MWCNT materials. The MWCNT-7, HT, and ND hydrodynamic diameter (DH) measurements were 411 ± 16, 499 ± 15, and 432 ± 18 nm, respectively. The zeta potential for MWCNT-7, HT, and ND were − 40 ± 1, − 51 ± 1, and − 49 ± 2 nm, respectively (Table 1). Suspension stability analyses found that all three particles significantly differed from each other in their stability over time in SAGM medium (*p* < 0.05) with a stability ranking of MWCNT-ND > HT > − 7 (Fig. 2). This indicated that SAECs exposed to MWCNT-7 experienced a larger deposited dose of MWCNT over time than the other two particle treatments. Similarly, MWCNT-7 showed significant less stability over time compared to MWCNT-HT and ND in DMEM medium (Fig. 2; p < 0.05), while MWCNT-HT and ND did not differ from each other. All MWCNT were more stable in serum-containing medium, DMEM, than SAGM suggesting lower deposited doses over time to BEAS-2B cells compared to SAECs.

### Partitioning of MWCNT material into cell nuclei

**Fig. 3:**
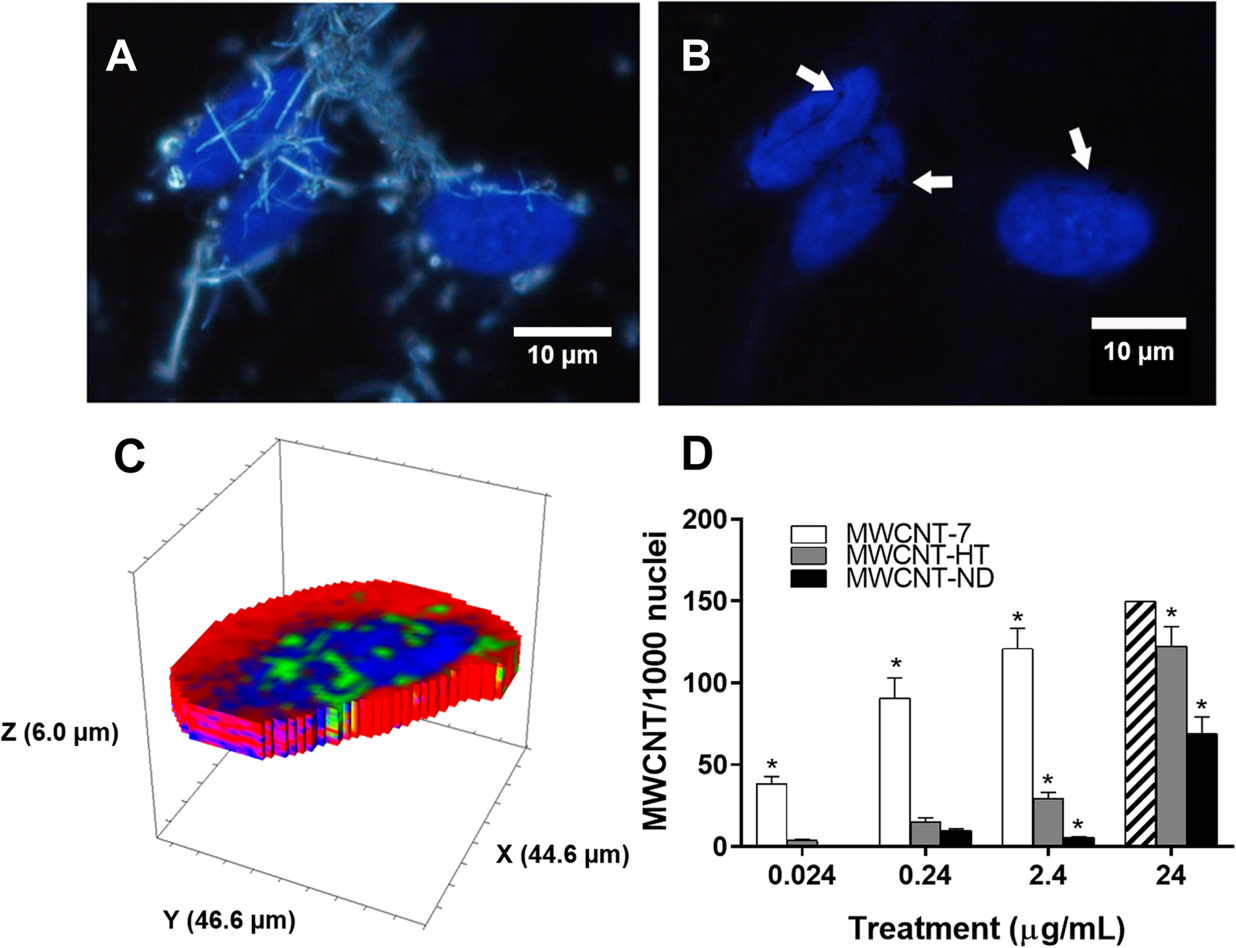
Each MWCNT material entered the nucleus of BEAS-2B after 24 h of exposure, but nuclear uptake was greater for MWCNT-7 than MWCNT-HT & ND. **a** Composite image of enhanced dark-field showing the MWCNT fibers in white and the blue fluorescent DAPI stained nuclei. Magnification bar is 10 μm. **b** Fluorescence only image of A showing areas where blue DAPI stain for DNA has been displaced by the MWCNT-HT material within the nucleus and, therefore, appears as a black hole (white arrows). Magnification bar is 10 μm. **c** A 3D rendering of a BEAS-2B cell exposed to MWCNT-HT for 24 h overlaid with Raman spectra. The red indicates silica material from the glass microscope slide, the blue indicates nuclear protein, and the green indicates MWCNT-HT material. This image shows the MWCNT-HT material throughout the entire nucleus. **d** MWCNT-7 white, MWCNT-HT gray, and MWCNT-ND black bars. Partition of MWCNT in the nucleus is given as the number of individual nanotubes per 1000 nuclei. MWCNT-7 partitioning into nuclei at the highest dose of 24 μg/mL were too numerous to accurately count as indicated by the hashed bar. For MWCNT-ND, partition into nuclei at the lowest dose of 0.024 μg/mL was zero. * indicates significantly different from counterpart MWCNT material, *p* < 0.05; ± SD

Quantification of nuclear uptake was measured in the BEAS-2B cells by Enhanced Darkfield Microscopy imaging. The data are reported as the number of single MWCNT within the nucleus per 1000 nuclei. All three MWCNT materials have a high lipid solubility and will freely partition into and/or across lipid membranes. Frequently, MWNCT were found on the outer surface of the nucleus (Fig. 3a) or within the nucleus (Fig. 3b & c). For each MWCNT material, the partitioning of individual MWCNT within the nucleus increased in a dose-dependent manner following 24 h of exposure (Fig. 3d). MWCNT-7 consistently demonstrated a higher partitioning of individual nanotubes into the nucleus compared to MWCNT-HT and ND. For example, 2.4 μg/mL exposure of either MWCNT-7, HT, or ND had, on average, 121, 30, and 6 single nanotubes per 1000 nuclei, respectively (Fig. 3d). Most notably, at the lowest dose of 0.024 μg/mL no MWCNT-ND were observed in the nucleus, whereas at the highest dose of 24 μg/mL the uptake of MWCNT-7 was so high an accurate measurement was unobtainable. The cellular localization of MWCNT was confirmed by Raman confocal imaging. Three-dimensional mapping by Raman confocal microscopy showed MWCNT material within the nucleus and dispersed throughout the nucleus (Fig. 3c) in MWCNT-exposed immortal and primary cells. Finally, MWCNT material within the nucleus of BEAS-2B was confirmed through transmission electron microscopy (TEM; Additional file 1: Figure S1).

### Cytotoxicity

**Fig. 4:**
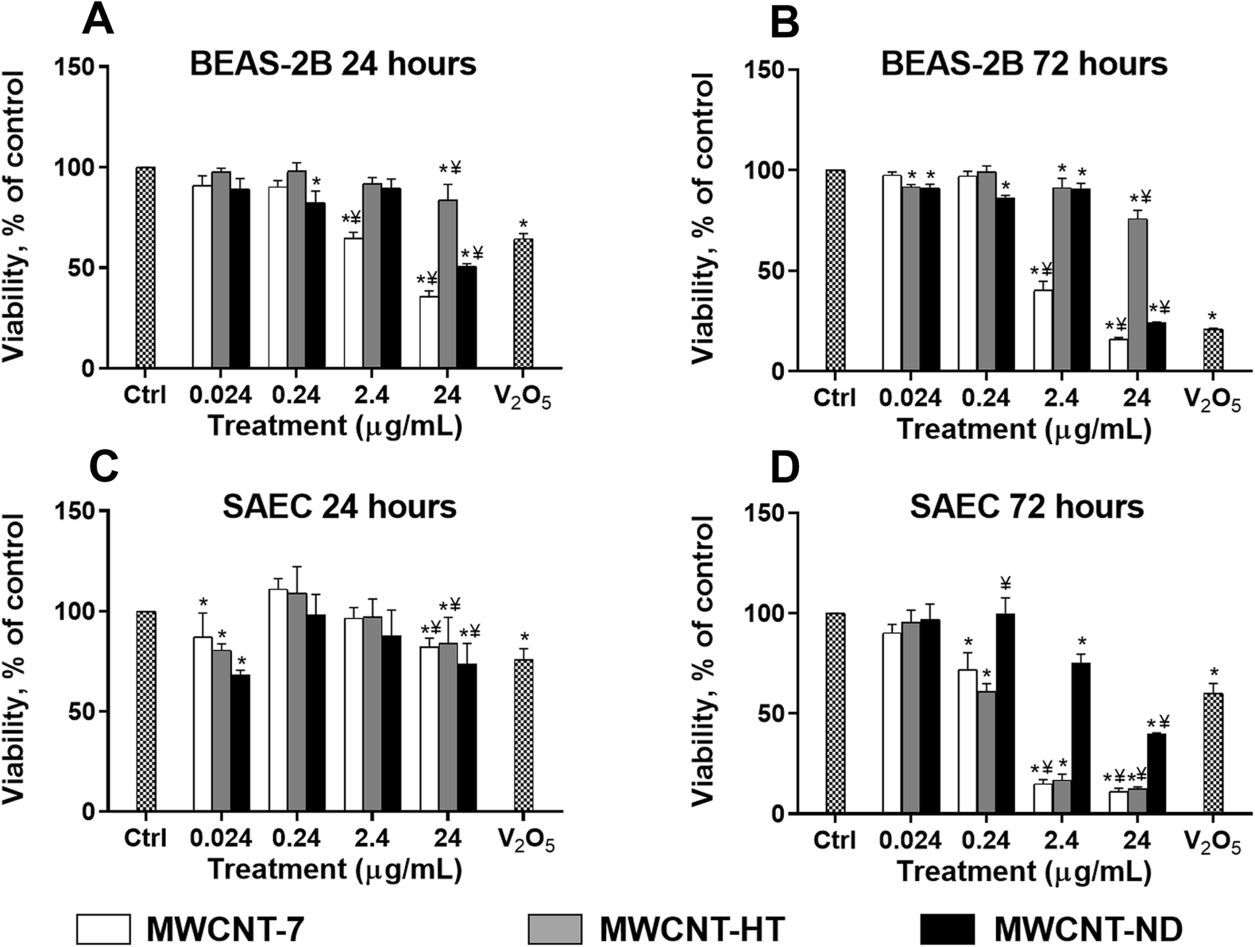
Cytotoxicity in BEAS-2B and SAEC after 24 and 72 h of exposure to each MWCNT material. **a**. BEAS-2B, 24 h. **b**. BEAS-2B, 72 h. **c**. SAEC, 24 h. **d**. SAEC, 72 h. MWCNT-7 white, MWCNT-HT gray, and MWCNT-ND black bars. V2O5 was used as a positive control, 0.316 μg/mL in BEAS-2B and 3.16 μg/mL in SAEC cell. * indicates significantly different from control, *p* < 0.05. ¥ indicates significantly different from other MWCNT materials at same dose, p < 0.05; ± SD

Cell viability following 24 and 72 h of exposure to each MWCNT material was measured in both cell types. In BEAS-2B, viability was reduced in a dose-dependent manner after exposure to each MWCNT material for 24 and 72 h with the longer exposure time producing a greater reduction of viability (Fig. 4a & b). Exposure to MWCNT-7 induced the greatest reduction in viability at either time point. In addition, exposure to 24 μg/mL of each MWCNT material produced significant cytotoxicity compared to control and each other (Fig. 4a & b). In SAEC, viability was reduced in a dose-dependent manner after exposure to each MWCNT material for 72 h with significant cytotoxicity after exposure at the 0.24, 2.4, and 24 μg/mL doses (Fig. 4d). Similar to MWCNT-exposed BEAS-2B cells, the MWCNT-7 material demonstrated the greatest reduction in viability and each MWCNT material produced significant cytotoxicity compared to control and each other at the highest two doses (Fig. 4d). However, exposure to only the 0.024 and 24 μg/mL doses of each MWCNT material for 24 h significantly reduced cell viability (Fig. 4c).

### Mitotic aberrations

**Fig. 5:**
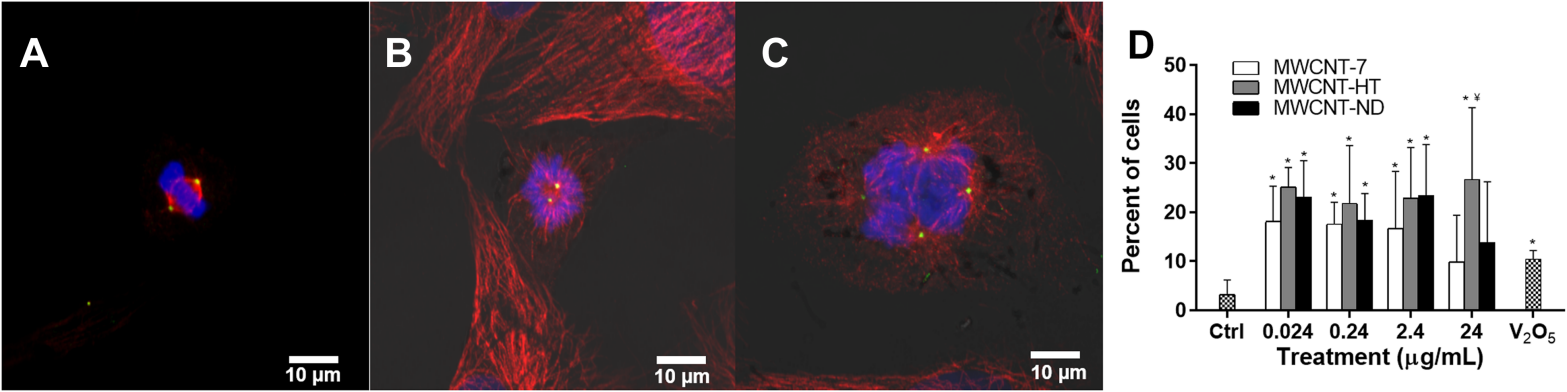
Mitotic spindle disruption and fragmented centrosomes were observed in BEAS-2B cells exposed to each MWCNT material. **a-c** DNA is blue, centrosomes are green, and mitotic spindle is red in a normal (**a**), monopolar (**b**), and multipolar (**c**) spindle morphology. **d** Centrosome fragmentation was significantly increased. MWCNT-7 white, MWCNT-HT gray, and MWCNT-ND black bar. V2O5 was used as a positive control, 0.316 μg/mL. Magnification bar is 10 μm. *indicates significantly different from control, *p* < 0.05. ¥ indicates significantly different from other MWCNT materials at same dose, p < 0.05; ± SD

The mitotic spindle of BEAS-2B fluorescently-labeled for DNA, β-tubulin, and pericentrin was analyzed using confocal microscopy to determine the effects of exposure to MWCNT-7, HT and ND on cell division. We observed a significantly greater percentage of mitotic spindle aberrations with exposure to each MWCNT material compared to control (Table 2). A mitotic spindle aberration is defined as a mono- or multipolar spindle morphology and/or fragmented centrosome (Fig. 5). Given the inherent cytotoxicity of MWCNT material, the percentage of dividing cells was measured and recorded as mitotic index. After 24 h in culture, 7% of control cells were dividing with 7% aberration. Compared to control, cells exposed to V_2_O_5_, a known mitotic spindle disrupter [37, 43], demonstrated significantly less divisions (3 ± 1%), yet greater mitotic spindle disruption (14 ± 1%) and centrosome fragmentation (10 ± 2) (Table 2 and Fig. 5d). Divisions were significantly reduced following exposure to 24 μg/mL MWCNT-7 and ND compared to control; indeed, only 2% of MWCNT-7 treated and 3% of MWCNT-ND treated cells were dividing. Therefore, the low and non-significant percentage of mitotic disruption observed following these exposures was reasonably due to cytotoxicity, which is evident in Fig. 4. Cells exposed to 0.024 and 0.24 μg/mL MWCNT-7, 0.24, 2.4 and 24 μg/mL MWCNT-HT, and 0.24 μg/mL MWCNT-ND had significant mitotic spindle disruption compared to control (Table 2). Observation of the mitotic spindle morphology demonstrated that both multi-and monopolar configurations were present, however the monopolar morphology predominated (Table 2).

**Table 2:** Mitotic aberrations in BEAS-2B cells exposed to MWCNT material

Treatment		Mitotic Index	Spindle Aberration	Monopolar	Multipolar	Centrosome Fragmentation
Control		7 ± 3	7 ± 3	2 ± 2	5 ± 1	3 ± 3
V_2_O_5_		3 ± 1*	14 ± 1	8 ± 2	6 ± 2	10 ± 2*
MWCNT-7 (μg/mL)	0.024	6 ± 1	17 ± 7*	10 ± 9	7 ± 3	18 ± 7*
	0.24	5 ± 3	17 ± 2*	10 ± 6	7 ± 5	18 ± 4*
	2.4	5 ± 3	7 ± 6	5 ± 4	2 ± 2	17 ± 12*
	24	2 ± 2*	9 ± 7	2 ± 4	4 ± 4	10 ± 10
MWCNT-HT (μg/mL)	0.024	6 ± 3	14 ± 7	6 ± 5	8 ± 4	25 ± 4*
	0.24	6 ± 3	20 ± 20*	12 ± 16	8 ± 5	22 ± 12*
	2.4	8 ± 5	20 ± 12*	15 ± 15	5 ± 3	23 ± 10*
	24	4 ± 1	18 ± 5*	14 ± 5	4 ± 1	27 ± 15*
MWCNT-ND (μg/mL)	0.024	6 ± 3	13 ± 9	7 ± 7	5 ± 2	23 ± 8*
	0.24	5 ± 4	24 ± 12*	15 ± 11	9 ± 3	18 ± 6*
	2.4	5 ± 3	15 ± 11	9 ± 11	6 ± 4	23 ± 10*
	24	3 ± 3*	6 ± 5	3 ± 6	3 ± 5	14 ± 12

This table includes the percentage of mitotic aberrations after exposure to MWCNT material in a dose response and 0.316 μg/mL V_2_O_5_, positive control, for 24 h. Spindle aberration is reported as the sum of the percentage of monopolar and multipolar mitotic spindles. *indicates significantly different from control, p < 0.05; ± SD

Centrosome fragmentation was significantly increased after exposure to 0.024, 0.24, and 2.4 μg/mL of each MWCNT material and 24 μg/mL of MWCNT-HT compared to control (Table 2 and Fig. 5d). Centrosome fragments were observed organizing into either bipolar, multipolar, or monopolar spindle morphologies (Additional file 2: Figure S2) that can progress through mitosis (Additional file 3: Figure S3A). However, centrosome fragmentation can also lead to severely disrupted mitotic spindle morphologies that cannot be classified (Additional file 3: Figure S3C & D). Misaligned DNA and catastrophic spindle morphologies were observed after exposure to each MWCNT material (Additional file 3: Figure S3B-D). Differential interference contrast imaging found each MWCNT material throughout the nucleus of exposed cells and demonstrated an affinity between MWCNT material and the spindle poles (Fig. 5c & c, Additional files 2: Figure S2 & Additional files 3: Figure S3).

### Chromosome analysis

FISH analysis of interphase cells for chromosomes 1 and 4 in SAEC demonstrated a significant percentage of loss and gain of chromosomes 1 and 4 (total aneuploidy) after exposure to each MWCNT material in a dose response (Table 3). After 24 h, the percentage of total aneuploidy in control was 9 ± 4% which is within the acceptable range established for clinical evaluation set forth by the American College of Medical Genetics (ACMG) and Witkor and associates [58–60]. When cells were exposed to V_2_O_5_, a known aneugenic substance, 41 ± 11% of total aneuploidy was observed. Exposure to the highest dose of 24 μg/mL MWCNT-7, HT, and ND induced significantly greater total aneuploidy (67 ± 3, 61 ± 7, and 56 ± 14%, respectively) compared to control. A 1000-fold reduction in dose of MWCNT-7, HT, ad ND produced 59 ± 13, 44 ± 14, and 48 ± 18% of total aneuploidy, respectively, which is greater than that in the positive control. When chromosomes 1 and 4 were analyzed individually, a statistically significant dose-dependent increase in aneuploidy was observed for each MWCNT material (Table 3). Total aneuploidy in cells exposed to 0.024 μg/mL and chromosome 1 aneuploidy in cells exposed to 0.24 μg/mL was significantly different between MWCNT-7 and HT material indicating a possible difference in effect due to physicochemical properties.

**Table 3:** Aneuploidy in SAEC cell exposed to MWCNT material

Treatment		Total Aneuploidy (%)	Chromosome1 (%)	Gain (%)	Loss (%)	Chromosome 4 (%)	Gain (%)	Loss (%)
Control		9 ± 4	7 ± 3	4 ± 2	3 ± 3	6 ± 3	4 ± 1	3 ± 2
V_2_O_5_		41 ± 11*	33 ± 13*	13 ± 4	20 ± 11	19 ± 9	9 ± 3	10 ± 6
MWCNT-7 (μg/mL)	0.024	59 ± 13*^+^	41 ± 5*	10 ± 6	31 ± 11^¥^	37 ± 19*	19 ± 24	18 ± 7
	0.24	59 ± 13*	45 ± 8*^+^	13 ± 10	32 ± 16^¥^	35 ± 17*	13 ± 13	21 ± 8
	2.4	61 ± 7*	45 ± 16*	7 ± 2	39 ± 18^¥^	40 ± 13*	9 ± 8	31 ± 13^¥^
	24	67 ± 3*	49 ± 7*	9 ± 2	40 ± 6^¥^	48 ± 3*^+^	8 ± 5	40 ± 8^¥^
MWCNT-HT (μg/mL)	0.024	44 ± 14*^+^	32 ± 5*	18 ± 10	14 ± 6	29 ± 13*	14 ± 8	15 ± 7
	0.24	45 ± 13*	31 ± 1*^+^	14 ± 3	17 ± 3	29 ± 16*	19 ± 16	10 ± 0
	2.4	51 ± 6*	38 ± 7*	14 ± 11	24 ± 13	29 ± 10*	15 ± 9	15 ± 7
	24	61 ± 7*	45 ± 6*	8 ± 6	36 ± 8^¥^	38 ± 8*	10 ± 7	28 ± 14^¥^
MWCNT-ND (μg/mL)	0.024	48 ± 18*	36 ± 8*	14 ± 8	21 ± 5	30 ± 15*	16 ± 9	15 ± 7
	0.24	46 ± 10*	33 ± 4*	9 ± 5	23 ± 7^¥^	29 ± 13*	17 ± 12	12 ± 2
	2.4	51 ± 6*	42 ± 7*	11 ± 10	31 ± 11^¥^	28 ± 13*	15 ± 5	13 ± 10
	24	56 ± 14*	42 ± 8*	16 ± 13	26 ± 8	34 ± 15*^+^	16 ± 15	18 ± 4

This table represents the percentage of aneuploidy in SAEC cells exposed to each MWCNT material and 0.316 μg/mL V_2_O_5_, positive control, for 24 h. Percentage of total aneuploidy is representative of aneuploidy of chromosomes 1 and 4 combined and based on the total number of cells analyzed. Percentage of aneuploidy was also separated by chromosome and a loss or gain of either chromosome 1 or 4 was also recorded as a percentage of total cells. * indicates significantly different from control, p < 0.05. ¥ indicates significantly different from counterpart MWCNT materials, p < 0.05. + indicates significantly different from each other, within treatment group, p < 0.05. ± SD

**Fig. 6:**
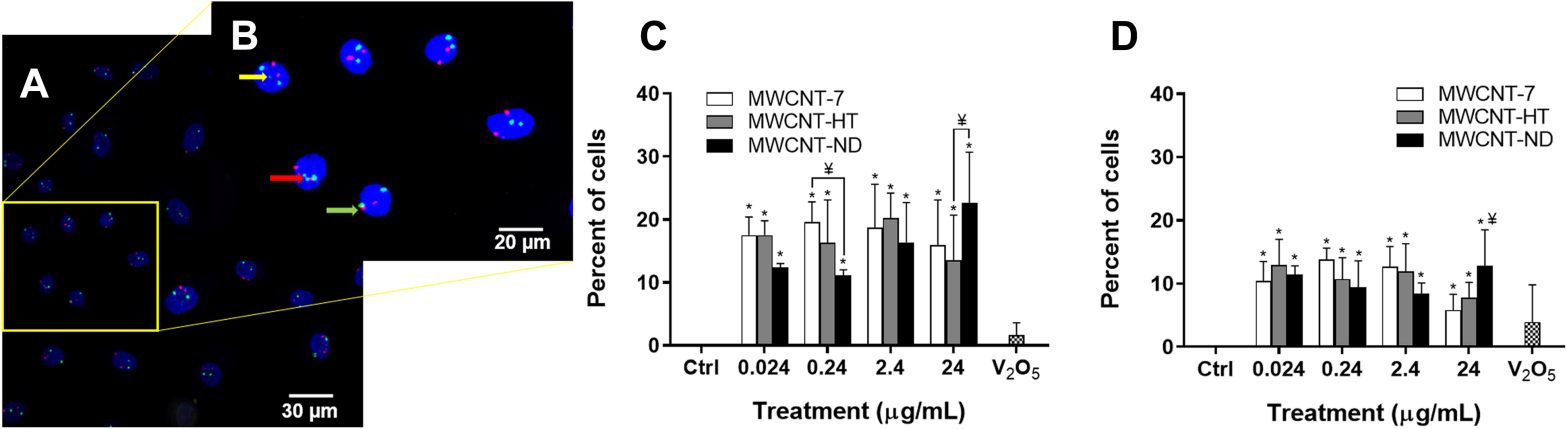
Fragmentation and translocation of the centromeres of chromosomes 1 & 4 in SAEC exposed to MWCNT material. **a** Nuclei of cells exposed to MWCNT-7 material are stained blue, chromosome 1 in red, and chromosome 4 in green. Magnification bar is 30 μm. **b** Region of interest, yellow arrow points to centromere fragment of chromosome 4. Red arrow points to chromosome 4 inserted into chromosome 1. Green arrow points to translocation between centromeres of both chromosomes. Magnification bar is 20 μm. **c** & **d**) Graphical representation of centromere fragmentations (**c**) and translocations (**d**). MWCNT-7 white, MWCNT-HT gray, and MWCNT-ND black bars. V2O5 was used as a positive control, 3.16 μg/mL. * indicates significantly different from control, p < 0.05. ¥ indicates significantly different from other MWCNT materials at same dose, p < 0.05; ± SD

FISH labeling specific to the centromeric regions of chromosomes 1 and 4 allowed for the analysis of chromosomal translocation as well as centromere integrity. Insertions and translocations (Fig. 6a & b) were observed in the nuclei of cells exposed to each dose of MWCNT-7, HT, and ND, regardless of nuclear uptake of the material (Fig. 3d). This is to be expected given that the nuclear membrane is degraded during cell division, therefore allowing MWCNT material within the cytosol to interact with nuclear material. A quantitative analysis demonstrated a significantly greater percentage of fragmentation (Fig. 6c) and translocation (Fig. 6d) after exposure to each MWCNT material for 24 h. The percentage of fragmentation following exposure to 0.024 and 0.24 μg/mL MWCNT-7 and ND was significantly different indicating a possible difference in effect regarding physicochemical properties (Fig. 6c). Additionally, the percentage of translocations following exposure to 24 μg/mL MWCNT-7 and ND was also significantly different further indicating that physicochemical properties affect centromere integrity (Fig. 6d). However, the lack of a dose-dependent response is most likely due to the inherent low mitotic index of the SAEC cell type following exposure to the rigid MWCNT material.

### Cell cycle analysis

Bivariate flow cytometry analyses of fluorescently-labeled DNA in BEAS-2B and SAEC exposed to each MWCNT material indicated genotoxicity through significant arrests in the cell cycle (Table 4).

**Table 4:** Cell Cycle Analysis

Cell Type	Exposure time (h)	Treatment	%G1	%S	%G2
BEAS-2B	24	Control	38 ± 2	23 ± 2	37 ± 3
		Arsenic	45 ± 3*	17 ± 2*	35 ± 2
		MWCNT-7	42 ± 4	33 ± 3*	25 ± 2*
		MWCNT-HT	36 ± 2	34 ± 5*^¥^	27 ± 4*
		MWCNT-ND	39 ± 2	28 ± 4^¥^	30 ± 3*
SAEC	24	Control	61 ± 2	29 ± 3	8 ± 1
		Arsenic	64 ± 3	25 ± 4*	9 ± 1
		MWCNT-7	76 ± 4*	10 ± 5*	10 ± 1
		MWCNT-HT	81 ± 1*^¥^	5 ± 1*^¥^	11 ± 1*
		MWCNT-ND	75 ± 1*^¥^	14 ± 1*^¥^	10 ± 1*
SAEC	72	Control	74 ± 5	15 ± 6	11 ± 1
		Arsenic	72 ± 2	7 ± 1*	18 ± 1*
		MWCNT-7	56 ± 2*^¥^	31 ± 3*	13 ± 1
		MWCNT-HT	70 ± 6	21 ± 6*^¥^	10 ± 1
		MWCNT-ND	68 ± 5*	21 ± 5*	11 ± 2

This table shows the mean percentage of cells in the G1, S, and G2 phase of the cell cycle measured via flow cytometry. The 24 h exposures used 24 μg/mL MWCNT doses whereas the 72 h exposure used 2.4 μg/mL MWCNT doses. BEAS-2B used 5 μM arsenic as a positive control whereas SAEC used 10 μM arsenic as a positive control. *Significantly different from control, p < 0.05 ¥Significantly different from other MWCNT materials at same dose, p < 0.05; ± SD

After 24 h, the BEAS-2B control cell cycle population demonstrated 38 ± 2, 23 ± 2, and 37 ± 3% of cells in the G1, S, and G2 phases of the cell cycle, respectively (Table 4). Exposure to 5 μM arsenic (positive control) demonstrated significantly less S phase cells (17 ± 2%) and significantly greater G1 and G2 phase cells (45 ± 3 & 35 ± 2%) compared to control. Exposure to 24 μg/mL MWCNT-7 and HT produced significantly more S phase cells (33 ± 3 and 34 ± 5%, respectively) and significantly less G2 phase cells (24.6 ± 2.0 and 26.8 ± 3.9%, respectively) compared to control. However, the MWCNT-ND at the same dose demonstrated a greater amount of G1 and S phase cells (28 ± 4 & 39 ± 2%, respectively) that were not significantly different from control, but significantly less G2 phase cells (30 ± 3%). These data indicate that 24 h of exposure to 24 μg/mL of each MWCNT material produced an arrest in G1/S and arsenic produced an arrest in G1 and G2 phases of the cell cycle in an immortalized cell.

Control cell cycle populations in SAEC after 24 h were 61 ± 2, 29 ± 3, and 8 ± 1% for G1, S, and G2 phases, respectively (Table 4). Exposure to 10 μM arsenic demonstrated significantly less S phase cells (25 ± 4%) compared to control. There were a greater amount of cells in the G1 and G2 phases, but there were not significantly different from control (25, 64 ± 3, & 9 ± 1%, respectively). However, exposure to 24 μg/mL of each MWCNT material demonstrated significantly less S phase and greater G1 and G2 phase cells compared to control (Table 2b). These data indicate that 24 h of exposure of primary cells to 24 μg/mL of each MWCNT material and 10 μM arsenic produced an arrest in G1 and G2 phases of the cell cycle.

After 72 h, the SAEC control cell cycle populations were 74 ± 5, 15 ± 6, and 11 ± 1% in the G1, S, and G2 phases, respectively (Table 4). Exposure to 10 μM arsenic demonstrated significantly less S phase cells (7 ± 1%) but greater G2 cells (18 ± 1%) compared to control. Exposure to 2.4 μg/mL of MWCNT-7 and ND material produced significantly more S phase cells (31 ± 3 and 21 ± 5%, respectively) and less G1 phase cells (56 ± 2 and 68 ± 5%, respectively) compared to control. However, the MWCNT-HT material’s effect on the amount of G1 phase cells was slightly attenuated while demonstrating significantly more S phase and less G2 phase cells (21 ± 6% & 10 ± 1%, respectively) compared to control (Table 4). These data indicate that 72 h of exposure of primary cells to 2.4 μg/mL of each MWCNT material induced an arrest in G1/S and 10 μM arsenic induced an arrest in G1 and G2 phases of the cell cycle.

### Clonal growth

**Fig. 7:**
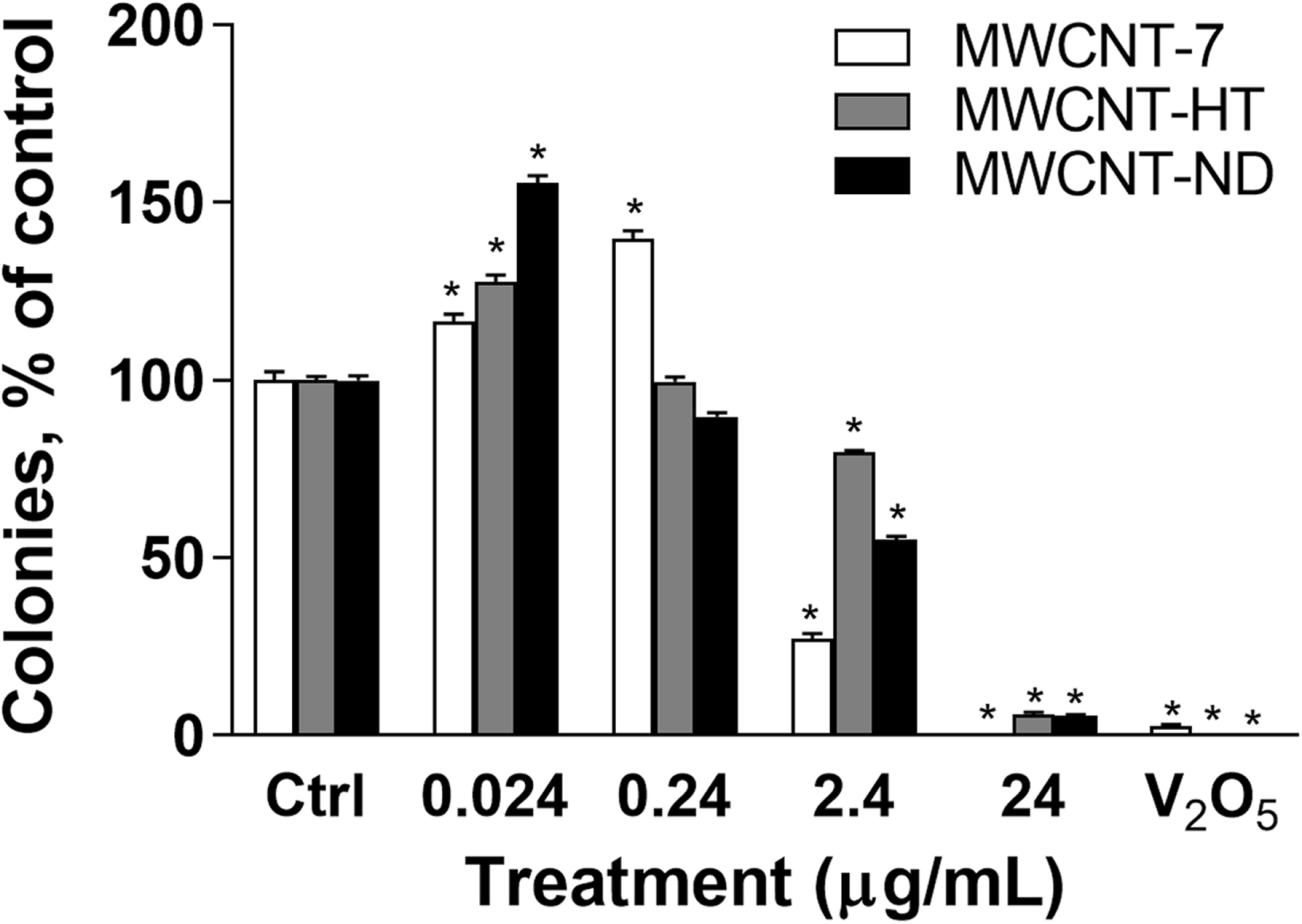
Clonal growth in SAEC exposed to each MWCNT material. MWCNT-7 white, MWCNT-HT gray, and MWCNT-ND black bars. V2O5 was used as a positive control, 3.16 μg/mL. * indicates significantly different from control, p < 0.05, ± SD

Exposure to each MWCNT material produced significant effects on clonal growth in SAEC. The percentage of colonies was significantly reduced from exposure to 24 & 2.4 μg/mL of each MWCNT material. The reduction in colony formation was reasonably due to cytotoxicity (Fig. 4c & d). However, clonal growth was significantly increased following exposure to 0.024 μg/mL of each MWCNT material and 0.24 μg/mL of MWCNT-7 (Fig. 7).

## Discussion

Carbon nanotubes have numerous applications in electronics, sports equipment, protective clothing, aerospace, fiber optics and molecular diagnostics [1]. This has led to an increased global production of MWCNT that is projected to reach 7000 tons by year 2025 in large part due to the manipulation of their unique physicochemical properties [61]. Although these characteristics present myriad opportunities for enhanced industrial applications, the risk to human health and lung disease has not yet been determined. Pristine MWCNT-7 carbon nanotube was designated as a Group 2B carcinogen; however, other forms of MWCNT were not classified due to insufficient data [3]. The novel studies described in this manuscript will help fill the gap and provide mechanistic evidence of the carcinogenicity of MWCNT-7 as well as MWCNT with varying physicochemical properties compared to the MWCNT-7.

Our data demonstrate that exposure to MWCNT-7, HT & ND material disrupts cellular division leading to predominantly monopolar mitotic spindles. The aberrant mitotic cells had fragmented centrosomes, and abnormal DNA alignment. Most notably, the data reported in this manuscript are the first to show fragmentation of the centromere, chromosomal translocations, and chromosomal insertions following exposure to carbon nanotubes. A quantitative analysis of chromosome aberrations in primary human cells determined that exposure to each of the MWCNT material produced significant cell cycle disruption, aneuploidy, centromere fragmentations, and centromere translocations at all doses that resulted in a loss of chromosomes 1 and 4. The data further demonstrated that MWCNT-HT & ND material led to the same type of mitotic spindle and DNA disruption as MWCNT-7 indicating that these physicochemical alterations do not affect the mechanism of genotoxicity. One month post-exposure, the primary SAEC exposed to the lowest dose of 0.024 μg/mL of each MWCNT material demonstrated increased proliferation in culture. These data indicate that each MWCNT material, regardless of physicochemical alteration, caused significant genotoxicity and are, therefore, potentially carcinogenic.

Although each MWCNT material produced similar and significant genotoxicity in two different human lung epithelial cell types, the incidence was consistently lower for the MWCNT-HT & ND materials. We believe this to be a function of dosimetry rather than the material’s interaction with cellular structures. As shown in Figs. 2 and 3, differences in the partitioning of individual nanotubes into cell nuclei and sedimentation rate have significantly affected the delivered dose. In vertebrate-derived cell types with an approximate doubling time of 24 h, the duration of cell cycle phases is as follows: mitosis, 30 min; G1, 9 h; S, 10 h; G2, 4.5 h. Since the nuclear membrane is known to retract into the endoplasmic reticulum at the onset of mitosis, it’s not likely that the delivered dose is significantly affected by differences in nuclear penetration. Rather, the physicochemical differences between MWCNT materials regarding sedimentation are a better indication since all experiments in the present study were conducted on single-layer adherent cells dosed via culture media. Indeed, the sedimentation assays performed in both SAGM (SAEC culture media) and DMEM (BEAS-2B culture media) over 48 h show significant differences in sedimentation rate with MWCNT-7 settling fastest followed by MWCNT-HT & ND, respectively. These data correspond well with the differences in genotoxicity incidence in the present study, however an extrapolation of these data to human dose and lung aerodynamics is beyond the scope of this paper.

We observed a cell cycle arrest at G1 and G2 phases in primary SAEC 24 h following exposure to each MWCNT material (Table 4). A G1 and G2 block in the cell cycle often occurs after DNA damage in primary cells with normal p53 function [62, 63]. Although previous investigations have demonstrated cell cycle disruption following MWCNT exposure of immortalized cells, these data are the first to show MWCNT-induced cell cycle disruption in a primary cell population [37, 43, 44, 56]. An arrest in the G1/S phase of the cell cycle indicates centrosomal damage [64–66]. Indeed, the results in this study demonstrated that each MWCNT material has been integrated into the spindle pole resulting in fragmented centrosomes (Fig. 5c & d), Additional files 2: Figure S2 & Additional files 3: Figure S3). Our previous analysis demonstrated incorporation of 10–20 nm diameter MWCNT material into the centrosome structure and centrosome fragmentation [37]. In the present study, exposure to each MWCNT material produced a significant increase in centrosome fragmentation (Table 2). Fragmented centrosomes can cluster to form into a functional bipolar spindle [67] (Additional files 2: Figure S2C & Additional files 3: Figure S3A). In this case, the DNA may be separated evenly, however the daughter cells will have an abnormal amount of centrosome material leading to a loss of spindle pole integrity in the subsequent division. Loss of spindle pole integrity can result in cell death or manifest as a multipolar division ultimately leading to aneuploidy [67] (Additional file 2: Figure S2C). Centrosomes that duplicate or fragment but do not separate into two poles will have a monopolar spindle morphology (Additional file 2: Figure S2B). Mitotic divisions with monopolar spindle morphology suffer from a failure to undergo cytokinesis resulting in polyploidy [67–69]. The resulting polyploid cells will form multipolar mitotic spindles in the next division. Fragmented centrosomes and aneuploid divisions in lung cancer correlate with an aggressive phenotype [66, 70].

We also observed misaligned DNA with chromatin outside of the mitotic spindle and separate from their fragmented centrosomes (Additional file 3: Figure S3C & SD) after exposure to each MWCNT material. These aberrations could be indicative of a single kinetochore attached to microtubules emanating from more than one spindle pole known as a merotelic attachment [70, 71]. Such aberrant attachments would not be detected by spindle assembly checkpoints throughout mitosis leading to propagation of genetically-altered cells [72]. Exposure to each MWCNT material resulted in significant aneuploidy in a dose-dependent manner with evidence of translocations including insertions (Fig. 6b) and significant proliferation observed through increased S-phase of the cell cycle (Table 4). Indeed, MWCNT material with a more ridged, needle-like structure was found to induce significant aneuploidy in the CHL/IU cell line that was greater than that caused by MWCNT with a more curved and agglomerated structure [73].

Each of the three MWCNT materials in the present study were observed within the DNA, the centrosomes, and the microtubules of the mitotic spindle apparatus as well as within the bridge of cytokinesis (Additional file 3: Figure S3A & SB), a unique event identified previously [74, 75]. Carbon nanotubes have been described as the nanotechnological counterpart to microtubules based on their rigidity, resiliency, and relative diameters [45]. These physical similarities may be the basis of the formation of functional biohybrids of carbon nanotubes with α- and β-tubulin during polymerization of the microtubules creating a more rigid mitotic spindle pole and, therefore, a reduction in spindle pole integrity [36]. Additionally, the alteration of MWCNT physicochemical properties could affect the direct interaction of carbon nanotubes with other nuclear structures including the microtubules, centrosomes, and DNA [17, 37, 50, 52, 53, 56].

Exposure to each MWCNT material in the present study produced significant centromere fragmentation and translocations regardless of physicochemical alteration or dose (Fig. 6). This type of chromosomal disruption has not been observed with the more flexible 15 nm diameter MWCNT [37] or 1 nm SWCNT [43]. Given the high frequency of centromere fragmentation, complex rearrangements such as translocations and insertions would be expected. Breaks within the centromere are common in human tumor populations with centromere fragmentation and, therefore, expected [76]. Since centromeric probes were used on interphase cells, the detection of other types of chromosomal rearrangements, such as dicentric chromosomes and/or telomeric translocations, was not possible. Chromosome segregation during mitosis relies upon a dynamic interaction between the kinetochore, a tubulin-attracting protein complex surrounding the centromere of the chromosome, and the microtubule [76]. During mitosis, the elasticity of the kinetochore microtubules prevents the shattering of the centromere during the separation of the chromosomes [77]. The formation of a microtubule/MWCNT hybrid during the polymerization of the kinetochore microtubules may result in a more rigid structure that fractures during the separation of the chromosomes in mitosis. Such catastrophic events have shown to correlate with later tumor stage, shorter patient survival, as well as resistance to treatment in breast and lung cancer patients [71, 78, 79]. Previous data demonstrated that pulmonary exposure to MWCNT-7 caused significant tumor promotion and tumor progression in the mouse lung [80], complete carcinogenesis in the rat lung [81], and malignant mesothelioma in the rat pleura [82]. Although the carcinogenicity of MWCNT-HT & ND have not been evaluated by a pulmonary route, previous studies have shown MWCNT 50 nm in diameter to be more carcinogenic than 20 nm diameter when administered by intraperitoneal injection [17, 20].

A recent investigation into the pathological significance of physicochemical properties of MWCNT in C57BL/6 N mice one year after a single intratracheal instillation of 54 μg/mouse indicated that materials with a smaller diameter and a more tangled structure led to greater histopathological changes such as macrophage infiltration, lymphocytic infiltration, and granuloma formation [83]. Since these responses are a necessary action of innate immunity, this could be interpreted as a protective effect leaving thicker, more needle-like MWCNTs, like the MWCNT-7, −HT, and -ND, in the lung to cause long-term damage. Chromosomal translocations, aneuploidy, and sustained proliferation are hallmarks of cancer and are important in tumor promotion by allowing preneoplastic cells to transform into frank neoplasms [84–86]. Although aneuploidy alone has not been shown to correlate with tumor formation in vivo, the combination of supernumerary centrosomes, spindle aberrations, and chromosomal disruptions has been shown to lead to advanced tumor stage [67, 71, 72, 87, 88]. The lowest genotoxic dose used in the current study of 0.024 μg/mL would require approximately 34 years of exposure at the NIOSH REL of 1 μg/m^3^ [89]. Demographic data on worker populations exposed to carbon nanotubes indicate a transient population and low risk of exposure [90]. However, recent exposure assessments have measured levels of carbon nanotubes in workplace air between 0.7 and 331 μg/m^3^ indicating that the in vitro dose used in the present study could be achieved in much less time [91–96]. Although epidemiological studies of MWCNT exposures in humans are extremely limited due to the long latency of lung cancer and the relatively short duration of exposure, several investigations in mice and rats have demonstrated increased mesothelioma and lung cancer after peritoneal and pulmonary exposure [17, 20, 80–82, 97, 98].

## Conclusion

Our previous investigations have shown that carbon nanotubes of 1–20 nm in diameter induce disrupted cell division and errors in chromosome number [37, 43, 44]. The data further indicated that diameter of the carbon nanotube was predictive of genotoxic potential with the 15 nm MWCNT in diameter [37] demonstrating greater genotoxicity than 1 nm SWCNT diameter [43, 44]. Exposure to MWCNT-7, HT, and ND with larger diameters ranging from 30 to 57 nm produced even greater genotoxicity as indicated by centromere fragmentation and translocations (Fig. 6). To our knowledge, this is the first investigation to report centromere fragmentation following exposure to any nanomaterial. The catastrophic genetic damage observed in this study may partially explain the basis of the potency of MWCNT-7 as a tumor promoter in the mouse and complete carcinogen in the rat [17, 20, 80–82]. Although physicochemical alteration of MWCNT reduced nuclear uptake of the material (Fig. 3d), all three materials caused the same type of genotoxic damage. Since in vitro genotoxicity is correlated with in vivo genotoxic response, these studies in primary human lung epithelial cells may predict the genotoxic potency in exposed human populations. The novel data presented herein indicate serious implications regarding the carcinogenicity of MWCNT-HT & ND materials and the risk assessment of “not classifiable” MWCNT material with varying physiochemical properties.

## Methods

### Materials

The MWCNT-7 material were a gift from Morinobu Endo and Shuji Tsuruoka (Shinshu University, Nagano, Japan), obtained through the Mitsui & Co., Ltd. (MWCNT-7, lot #05072001 K28) and previously characterized for length and diameter [55]. The MWCNT-HT and ND material are derivate of the MWCNT-7 material and were a gift from Mauricio Terrones (Pennsylvania State University, College Park, PA).

### Characterization

#### Length and diameter

A protocol was established for the measurements of diameter and length of raw MWCNT material using an SEM/Scanning Transmission Electron Microscope (STEM, S-5500 ultrahigh resolution SEM with STEM capabilities, Hitachi High Technologies America Inc., Schaumburg, IL 60173). The samples were prepared by adding a portion of the raw MWCNT material into a glass vial with isopropanol and sonicating for approximately 5 min to produce a well dispersed sample. A TEM grid (200 mesh Cu grid coated with carbon, SPI Supplies, West Chester, PA) was then dipped into the suspension and allowed to air dry. Without further coating, the material was examined by SEM. Initially a low magnification was used (~ 5-10kX) to locate fibers to measure. Once a fiber was located the magnification was increased appropriately to measure width and length. Using the measuring tools of the electron microscope’s software, straight lines were manually drawn to connect the desired distances to be measured. For length measurements, the longest straight line was drawn between two extremities of a fiber without following the curvatures of the fiber. For the width, measurements were taken by drawing a straight line of the distance perpendicular to the fiber’s walls. A minimum of 200 individual MWCNT structures were measured for each sample.

#### Purity

Scanning transmission electron microscopy (STEM) with energy dispersive X-ray spectroscopy (EDS) was used to qualitatively assess the purity of the three MWCNT materials by identifying the presence or absence of residual catalyst material in the material. Bright-field and dark-field electron microscopy were used to identify the catalyst material and EDS was used to confirm the elemental composition. High-resolution bright-field images were collected at an accelerating voltage of 200 kV (Hitachi HD-2300A STEM, Hitachi High Technologies America, Schaumburg, IL 60173). EDS spectra were collected to confirm the presence of Fe-rich catalyst material (Bruker Quantax, Bruker Nano Analytics, 12,489 Berlin, Germany). Inductively coupled plasma mass spectrometry (ICP-MS) was used to quantitatively measure residual metal contaminants in each MWCNT material. MWCNT samples were prepared and digested in triplicate. Dry MWCNT material was suspended in H_2_0 at a concentration of 1.0 mg/mL, vortexed for 10 s, and aliquoted (200 μL) into 40 mL PFTE digestion tubes containing 9 mL of ultrapure HNO_3_ and 1 mL of ultrapure H_2_O_2_. Tubes were capped and microwave-digested for 30 min at 200^o^ C. After cooling, digested samples were decanted into 50 mL polycarbonate centrifuge tubes, the digestion tubes were rinsed twice with 10 mL of H_2_O, and the rinses were added to the 5 mL polycarbonate tubes. The volume was adjusted to 40 mL with H_2_O. Samples were analyzed for ^52^Cr, ^58^Fe, ^62^Ni, and ^59^Co against certified reference standards using a Nexion inductively-coupled plasma mass spectrometer (Perkin-Elmer, Norwalk CT).

#### Suspension properties

Hydrodynamic diameter (D_H_) of each material was measured using photon correlation spectroscopy (PCS). Zeta potential was determined for each material suspended in water using laser Doppler electrophoresis [57]. All measurements were performed at 25 °C with a 633 nm laser at a 90° scattering angle (Zeta-sizer Nano ZS90, Malvern Instruments, Worcestershire, UK). The analyses were performed assuming a medium refractive index of 1.332, viscosity of 0.890 cP, dielectric constant of 78.3, and Smoluchowski approximation, f(κa) value of 1.5. Each suspension of MWCNT material was subject to ultrasonic agitation using a probe sonicator (XL 2000, QSonica, Newtown, CT) fitted with a 3-mm titanium probe tip. The delivered energy, as verified calorimetrically [99], was 27,600 J per sample. Distilled and deionized water that was passed through a 0.025 μm pore-size membrane (Anotop 25, Whatman International Ltd., Maidstone, England) was used to dilute each sample prior to analysis. Suspension stability index (SSI) analysis was conducted on each particle for each cell culture medium based on previously described methods [63]. Briefly, MWCNT suspensions in DM were sonicated, diluted to 0.1 mg/mL in each culture medium, immediately placed into cuvettes, and were assayed for absorbance at 325 nm, 500, and 550 nm on an Evolution 300 spectrophotometer with VisionPro software (ThermoScientific) at each hour over a 48 h period. Medium blanks were used to correct absorbance values for changes in absorbance over time for each medium. Three independent experiments were run. All data were normalized to 1 at zero hr. Quartic curve plots and regression analyses were conducted in SAS JMP v13.2. Parameter comparison and curve equivalence analyses (α = 0.05) were conducted to determine differences in SSI over time between MWCNT particles.

### Cell culture

Two pulmonary epithelial cell types were used in the present study. All cells were maintained at 37 °C and 5% CO_2_ with standard aseptic procedures. Immortalized human bronchial epithelial cells (BEAS-2B, ATCC, Manassas, VA) of less than 10 passages upon arriving in our laboratory were used to examine cytotoxicity, nuclear uptake, cell cycle arrest, mitotic aberrations, centrosome integrity, and spindle pole integrity. BEAS-2B were cultured in Dulbecco’s Modified Eagle Medium (DMEM) media supplemented with 10% (v/v) serum (Invitrogen, Grand Island, NY) and 1% (v/v) antibiotic-antimycotic (Corning, Corning, NY). Primary small airway respiratory epithelial cells (SAEC; Lonza, Walkersville, MD) from a non-smoking human donor were used to examine cytotoxicity, nuclear uptake, cell cycle arrest, aneuploidy, and clonal growth. The normal karyotype of the primary cells was essential for the examination of aneuploidy. The SAEC were cultured following manufacturer’s directions and using Cabrex media (Lonza, Walkersville, MD). Epithelial phenotype was identified in both cell types through EM analysis of stained cytokeratin 8 and 18 (data not shown) [37].

### Treatment protocol

#### Preparation of materials

Stock MWCNT material was subjected to 4–6 h of ultrasonic agitation over ice using a 3 mm titanium probe tip sonicator (Sonics and materials, Inc., Newtown, CT) set to 8 kHz for even dispersion in water. Just prior to use, the stock suspensions were dispersed similarly for one minute with a 10 s pulse in order to avoid an increase in temperature. Additionally, the media suspension containing the appropriate volume of stock MWCNT material was sonicated for 10 s before application to cell surface. Vanadium pentoxide (V_2_O_5_, sigma, St. Louis, MO) was suspended in dH_2_O and sonicated in a water bath (Branson 2510, fisher, Pittsburgh, PA) cooled with ice for 30 min immediately prior to addition to culture media. Sodium arsenite (arsenic, sigma, St. Louis, MO) was dissolved in dH_2_O

#### Cellular exposures

The BEAS-2B and SAEC were seeded in parallel culture dishes according to assay protocol. MWCNT doses were based on mass per volume of culture media (μg/mL) and also reported as mass per culture surface area (μg/cm^2^). Cells were exposed to MWCNT material suspended in appropriate culture media for either 24 or 72 h depending on assay requirements. Three independent experiments were performed for each assay.

### MWCNT material in cell nuclei

Confocal Raman spectroscopy was used to determine nuclear uptake and spatial orientation of each MWCNT material. Both BEAS-2B and SAEC were grown on glass chamber slides until 70% confluence and exposed to 24 μg/mL (4.2 μg/cm^2^) MWCNT-7, HT or ND for 24 h. After exposure cells were washed twice with phosphate buffered saline (PBS), fixed with 100% ethanol, and analyzed. The spectra of the MWCNT reference materials were generated using a Horiba LabRAM HR (Horiba Instruments, Edison, NJ, USA), equipped with an optical microscope, a 1024 × 256 pixel, Synapse CCD detector, a 600 grooves/mm grating, and a 473 nm argon laser. The parameters used to obtain the spectral data were as follows: 100 μm pinhole, 100x objective, a neutral density filter that attenuated all but 10% of the laser power, which resulted in a laser power at the sample of 286 μW, and two accumulations of each spectrum, collected for 5 s each, that were averaged together. A Raman map was generated to permit the analysis of a larger area containing the MWCNT material, and the resultant spectral data acquired from twenty different locations was baseline-corrected and averaged using the Horiba LabSpec 6 software package.

Cells were identified through brightfield imaging and Raman mapping of the cells was performed using a classical least squares (CLS) analysis for silica (glass slide), cellular protein, and MWCNT material using basis spectra. 3D renderings were produced using this data to determine the distribution of the MWCNT material within the nucleus. Raman spectroscopy was performed using an exposure time set to 1 s × 2 accumulations per pixel. The mapped areas were approximately 50x50x10 (XxYxZ) μm with a mapping step size set to 1 μm. Horiba LabSpec v6 software was used for data reduction and analysis.

Enhanced dark-field light microscopy was used to measure the partitioning of MWCNT into the nucleus of BEAS-2B cells. MWCNT have dimensions less than the wavelength of light, have closely packed atoms, and typically have a refractive index significantly different from that of biologic tissues and/or mounting medium. These factors cause nanoparticles, in general, and MWCNT, specifically, to be efficient light scattering structures. The enhanced dark-field microscope images only the light scattered by structures in the optical path. Typical biologic tissues such as cells and cell nuclei, and even the mounting media, produce minimal scattered light, and produce images in the enhanced dark-field microscope which are orders of magnitude lower in intensity than MWCNT. These characteristics which produce significantly greater scattering of light by nanoparticles produce images in which nanoparticles stand out with large, bright intensity compared to the surrounding biologic tissues that do not significantly scatter light. The significant intensity of scattered light from nanoparticles imaged by the enhanced dark-field microscope also produces a bright envelope or halo of scattered light about the outer edges of the nanoparticles which is significantly brighter than adjacent tissue that does not scatter light. Because of the significant scattering of light by nanoparticles, the enhanced dark-field microscope is able to detect nanoparticles in tissues and sections which could not otherwise be detected by a standard light microscope. The theory and application of this microscopy method to detect numerous types of nanoparticles (particles with dimensions less than 100 nm) in a variety of nanoparticle studies are described in detailed elsewhere [100].

For enhanced dark-field microscopy in the present study, the cells were grown to 70% confluence on glass chamber slides (Nunc™ Lab-Tek™ II, Waltham, MA), serum starved for 24 h, and exposed to 0.024, 0.24, 2.4 and 24 μg/mL (0.0042, 0.042, 0.42 and 4.2 μg/cm^2^) of each MWCNT material for 24 h. Cells were washed twice with PBS and fixed with 100% ice cold methanol. After fixation, nuclear content was fluorescently stained with DAPI (Vectashield, Vector Laboratories, Burlingame, CA) and individual nanotubes were counted using a high signal-to-noise, enhanced dark-field based illumination optics adapted to an Olympus bX-41 microscope (CytoViva, Auburn, AL 36830). Cells over five slides were counted per treatment. For counting of the partitioning of MWCNT into nuclei each cell nucleus of the slide was examined under enhanced dark-field illumination and any associated individual nanotubes were identified. Each individual nanotubes was then examined to determine if it was potentially partitioned within the cell nucleus by focusing from the top of the cell nucleus to the bottom. To be considered as being within the cell nucleus, the focusing over the entire cell nucleus had to demonstrate that the optical section(s) imaging the nanotube in the nucleus were sandwiched by optical slices above and below that were purely of cell nucleus and did not contain the individual nanotubes. The microscope was then re-focused on the nanotube in the nucleus and the microscope illumination was switched to fluorescent illumination of the DAPI nuclear stain. The nuclear partitioning of the individual nanotube was confirmed by the absence of the DAPI staining of nuclear material where the nanotube was located. MWCNT contained within the cell nucleus was reported as a mean ± SD of individual MWCNT in the nucleus per 1000 nuclei.

The presence of MWCNT material within the nucleus was confirmed by analyzing BEAS-2B cells exposed to 2.4 μg/mL MWCNT-7 for 24 h via transmission electron microscopy (TEM). The methods used for TEM sample preparation were similar to those previously followed [101]. Briefly, cells were fixed in 2% glutaraldehyde in sodium phosphate buffer, pH 7.2, for 2 h, post-fixed in osmium tetroxide, dehydrated through an ethanol series, and embedded in Spurr’s resin (Sigma, St Louis, MO). Silver-gold sections were stained in 2% aqueous uranyl acetate and Reynolds’ lead citrate, observed using a JEOL 1200 EX electron microscope, and recorded digitally.

### Cytotoxicity

Cytotoxicity of each MWCNT material was measured in both cell types. Cells were seeded in flat-bottom 96 well plates (Becton Dickinson Franklin Lakes, NJ) and exposed to 0.024, 0.24, 2.4 and 24 μg/mL (0.015, 0.15, 1.5 or 15 μg/cm^2^) of each MWCNT material for either 24 or 72 h. A 0.316 or 3.16 μg/mL or (0.2 or 2 μg/cm^2^) dose of V_2_O_5_ was used as positive control in the BEAS-2B or SAEC, respectively. Each treatment was measured in triplicate and the assay was repeated three times for each cell type. Cytotoxicity was assessed using the alamarBlue cell viability assay protocol following manufacturer directions (Invitrogen, Carlsbad, CA). Fluorescence was measured using a fluorescent spectrophotometer (LS50B, Perkin Elmer, Bridgeville, PA) with a 570 nm excitation and 585 nm emission wavelength. The fluorescence intensity was measured for each well. Cell viability is equivalent to a reduction in fluorescence intensity and was reported as a mean ± SD across all three experiments normalized to control.

### Mitotic aberrations

Laser scanning fluorescent confocal microscopy with differential interference contrast was used to analyze mitotic aberrations after exposure to each MWCNT material in the BEAS-2B cell (LSM 710, Carl Zeiss MicroImaging Inc.,Thornwood, NY). The relatively high mitotic index of the BEAS-2B cell type allows for sufficient examination of dividing cells. Cells were seeded on glass chamber slides (Nunc™ Lab-Tek™ II, Waltham, MA) until 70% confluence and exposed to 0.024, 0.24, 2.4 and 24 μg/mL (0.0042, 0.042, 0.42 and 4.2 μg/cm^2^) of each MWCNT material or 0.316 μg/mL (0.06 μg/cm^2^) V_2_O_5_ for 24 h.

After exposure, cells were washed twice and fixed with 100% methanol at 4 °C (Fisher Scientific, Waltham, MA). Dual chambers were prepared for each dose. The cells were stained for mitotic aberration analysis via fluorescent labeling of the DNA and immunofluorescent labeling of the mitotic spindle and centrosomes. The DNA was fluorescently labeled using DAPI (Vectashield, Vector Laboratories, Burlingame, CA). The β-tubulin of the mitotic spindle was labeled using a rabbit anti-β-tubulin primary antibody (Abcam, La Jolla, CA, USA) and goat anti-rabbit IgG secondary antibody conjugated with rhodamine red (Invitrogen, Carlsbad, CA). The centrosomes were labeled using mouse anti-pericentrin primary antibody (Covance, Austin, TX, USA) and goat anti-mouse IgG antibody conjugated with Alexa 488 (Invitrogen, Carlsbad, CA). Cells were examined and divisions were analyzed by photographing serial slices through the z-plane based on the depth of the cell and optical properties of the stain (Zen, Carl Zeiss MicroImaging Inc., Thornwood, NY). A minimum of 50 mitotic cells of good centrosome and mitotic spindle morphology were analyzed for each dose. Three independent experiments were conducted for a total of 150 cells.

Quantitative analysis of aberrant mitoses was based on spindle morphology; a mitotic cell with monopolar or multipolar spindle morphology was considered aberrant. Aberration was reported as a percentage of total mitotic cells analyzed for each dose across all three experiments. Centrosome and spindle pole integrity were assessed quantitatively. The association between MWCNT material and the labeled nuclear structures was also examined qualitatively by overlaying the fluorescent images with the differential interference contrast filter. The mitotic index is equivalent to the percentage of mitotic divisions in 100 cells per treatment.

### Chromosome analysis

Laser scanning confocal fluorescent microscopy imaging of fluorescently-labeled centromere of chromosomes 1 & 4 in SAEC was used to determine aneuploidy and centromere fragmentation after exposure to each MWCNT (LSM 710, Carl Zeiss MicroImaging Inc., Thornwood, NY). Chromosomes 1 & 4 were pragmatically chosen due to their size and labeling efficiency. Cells were seeded on glass chamber slides (Nunc™ Lab-Tek™ II, Waltham, MA) until 70% confluence and exposed to 0.024, 0.24, 2.4 and 24 μg/mL (0.0042, 0.042, 0.42 and 4.2 μg/cm^2^) of each MWCNT material or 3.16 μg/mL (0.06 μg/cm^2^) V_2_O_5_ for 24 h. After exposure, cells were washed twice and fixed with a 3:1 (v/v) mixture of methanol and acetic acid (Fisher Scientific, Waltham, MA). Chromosomes 1 and 4 were labeled via fluorescence in situ hybridization (FISH) of centromeric DNA (Abbott Molecular, Des Plaines, IL) and cells were fluorescently counterstained with DAPI (Vectashield, Vector Laboratories, Burlingame, CA) for chromatin content. Each batch of FISH probes were tested on normal human metaphase spreads isolated from normal human lymphocytes for a bright FISH signal at the correct location on the correct chromosome. The SAEC cell type has a normal karyotype, therefore chromosome enumeration for quantitative analysis of aneuploidy is possible. Cells were examined and scored according to the most stringent guidelines available ACMG [59]. Cells with three or greater than four signals for either chromosome were recorded as a gain; cells with less than two signals of either chromosome were recorded as a loss. Cells with exactly four signals were considered in active synthesis and excluded from the analysis. Only cells with good morphology were included in the analysis; cells with obvious signs of necrosis, apoptosis, and decondensed centromeres were not scored [102]. Use of centromeric probes allowed for a quantitative analysis of aneuploidy, centromere fragmentation, and translocations between chromosomes 1 and 4. Insertions between these two chromosomes, an extremely rare event, were noted but not quantitated. A fragment was determined if it was 1/3 or less the size of the normal centromere signal within that same cell. Translocations in interphase cells are identified by the presence of two fluorescent signals less than one signal distance apart. Only signals less than one signal distance apart that also had overlapping pixels were labeled positive for insertion/translocation, accordingly. A minimum of 100 interphase cells with satisfactory fluorescent signal were analyzed for each dose. Three independent experiments were performed for a total of at least 300 cells included in the analysis. Slides were coded and scored by three independent investigators. Aneuploidy was reported as a mean ± SD of the percentage of cells with either a gain or loss for each dose across all three experiments.

### Cell cycle analysis

Bivariate flow cytometry using the Click-iT EdU Alexa Fluor 647 flow cytometry assay kit (Molecular Probes, Eugene, OR) with 7-aminoactinomycin D (7-AAD, Invitrogen, Carlsbad, CA) allows for a more accurate analysis of the cell cycle compared to single-color methods. EdU (5-ethynyl-2′-deoxyuridine), a nucleoside analog of thymidine, is incorporated into DNA during the S phase of the cell cycle and covalently-labeled with Alexa Fluor 647 via a click chemistry reaction between an azide in the fluorophore and an alkyne within the EdU. The 7-AAD fluorophore is incorporated into the DNA of all fixed cells thereby staining for G1 and G2 phases of the cell cycle.

BEAS-2B and SAEC cell types were seeded in T25 flasks (Falcon, Corning, NY) until 70% confluence. BEAS-2B cells were exposed to 24 μg/mL (2.88 μg/cm^2^) of each MWCNT material and 5 μM arsenic for 24 h. EdU was applied after 22 h of exposure to allow for incorporation into the DNA. Cells were washed twice with PBS (Gibco, Waltham, MA) and 0.25% (v/v) trypsin in EDTA (Gibco, Waltham, MA) was used to remove cells from the flask surface. Two exposures were analyzed for the SAEC cell type requiring separate methods. First, SAEC cells were exposed to 10 μM arsenic or 24 μg/mL (2.88 μg/cm^2^) MWCNT material for 24 h. EdU was applied after 12 h of exposure. Cells were washed twice with PBS and removed from the flask with 0.25% (v/v) trypsin in EDTA (Lonza, Basel, Switzerland). Second, SAEC cells were exposed to 10 μM arsenic or 2.4 μg/mL (0.288 μg/cm^2^) MWCNT material for 72 h. EdU was applied after 12 h of exposure. Cells were washed twice with PBS and fresh media was applied for a 24 h recovery period. Each treatment was performed in triplicate. Cells were stained according to manufacturer’s instructions and run through a flow cytometer (LSR II, BD Biosciences Immunocytometry Systems, San Jose, CA). Ten thousand events were collected and the dual-labeled fluorescent DNA content was analyzed (FlowJo v10, FlowJo, Ashland, OR). Gating was set to exclude debris, non-cellular material, and doublets. The percentage of cells in G1, S, and G2 phases of the cell cycle were determined via manual gating of the bivariate analysis of the two fluorescent signals and reported as a mean ± SD across all experiments.

### Clonal growth

Enumeration of SAEC colonies was used to determine the clonal growth after exposure to each MWCNT. Cells were seeded in T25 flasks (Falcon, Corning, NY) until 70% confluence and exposed to 0.024, 0.24, 2.4 and 24 μg/mL (0.00288, 0.0288, 0.288, and 2.88 μg/cm^2^) of each MWCNT material or 3.16 μg/mL (0.4 μg/cm^2^) V_2_O_5_ for 24 h. After exposure cells were washed twice and removed from the flask surface with 0.25% (v/v) trypsin in EDTA (Lonza, Basel, Switzerland). Cells were reseeded in 6-well flat bottom plates (Falcon, Corning, NY) at 500 cells/well to allow for clonal growth from a single cell. Colonies were grown for one month and stained with a 10% (v/v) solution of crystal violet in neutral buffered formalin (Sigma, Saint Louis, MO) to preserve and identify clonal morphology. A stereo microscope (SZX12, Olympus, Shinjuku, Japan) was used to count the colonies in each of the six wells. The mean ± SD of colonies was calculated and reported as a percentage of control.

### Statistical analysis

All analyses were performed using SAS/STAT (Version 9.4) for Windows, and JMP version 12 (SAS Institute, Cary NC). Data were analyzed using appropriate linear models including one-way and two-way analysis of variance (ANOVA). Some experiments were performed using a randomized blocks design and mixed-model ANOVAs were utilized to include block as a random factor. The assumptions of the models such as homogeneity of variance were assessed by inspection of residual plots. For some variables a log transformation was utilized to reduce heterogeneous variances. All differences were considered statistically significant at *p* < 0.05.

## Additional files

Additional file 1: **Figure S1.** TEM of BEAS-2B cell exposed to 2.4 μg/mL MWCNT-7 for 24 h. The nuclear envelope is indicated by the black arrows. Some of the MWCNT that are enclosed in the cell cytoplasm are indicated by green arrows. Several of the indicated MWCNT at the top of the micrograph (larger green arrows) appear to be within membrane bound vesicles while other MWCNTs within the cell cytoplasm (small green arrows) at the bottom of the micrograph are not membrane bound. The single red arrow indicates a MWCNT within the nucleus. The MWCNT within the nucleus is not bound by a lipid membrane. Magnification bar is 2.5 μm. (TIF 948 kb)

Additional file 2: **Figure S2.** Fragmented centrosomes cluster into one pole in BEAS-2B cells exposed to each MWCNT material. A-C) DNA blue, centrosomes are green, and mitotic spindle is red. A) MWCNT-HT, bipolar spindle. B) MWCNT-7, monopolar spindle. C) MWCNT-ND, multipolar spindle. White arrows point to clusters of fragmented centrosomes. Magnification bar is 10 μm. (TIF 1192 kb)

Additional file 3: **Figure S3.** MWCNT can interfere with spindle attachment to the centromere to produce supernumerary centrosomes, misaligned DNA, and centrosome fragmentation that can be so great a normal mitotic spindle cannot be formed in BEAS-2B cells exposed to MWCNT material for 24 h. A-D) DNA blue, centrosomes are green, and mitotic spindle is red. A) MWCNT-ND; supernumerary centrosomes. B) MWCNT-HT, C & D) MWCNT-7; misaligned DNA and catastrophic spindle morphology. White arrows point to MWCNT material within the bridge of cytokinesis (A & B) or MWCNT interacting with the DNA, centrosomes, and mitotic spindle (C & D). Magnification bar is 10 μm. (TIF 1533 kb)

## Data Availability

The datasets used and/or analyzed during the current study are available from the corresponding author upon reasonable request.
