# Supplementary figures and images for: Mitsui-7, heat-treated, and nitrogen-doped multi-walled carbon nanotubes elicit genotoxicity in human lung epithelial cells
Source: Part Fibre Toxicol. 2019 Oct 7;16:36. doi: 10.1186/s12989-019-0318-0 (PMC6781364; doi:10.1186/s12989-019-0318-0)

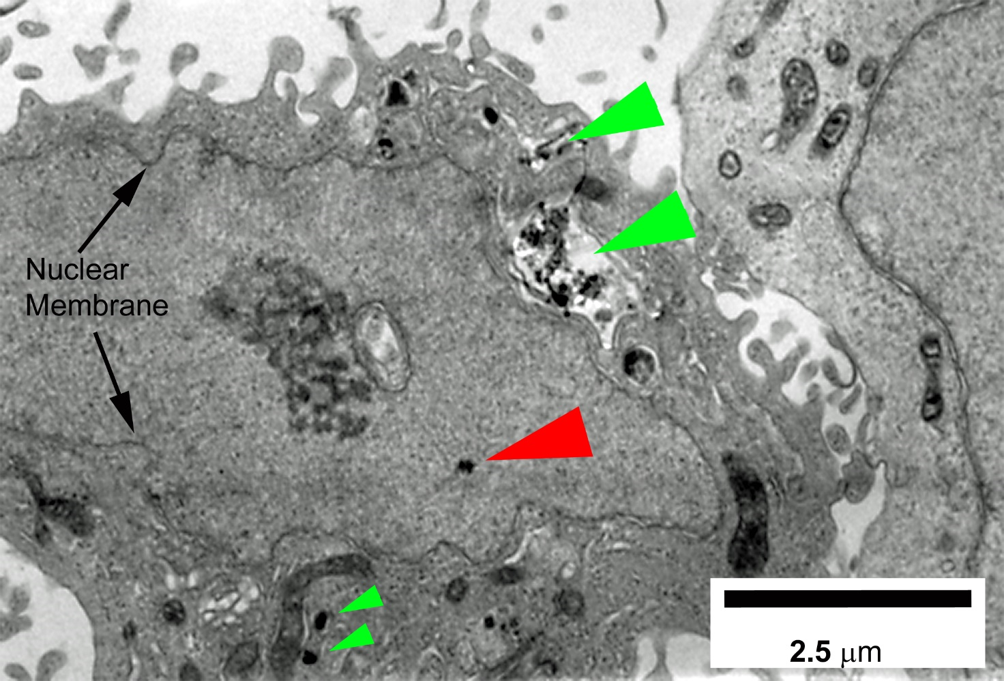

Supplement: Supplementary file 1 — Figure S1. TEM of BEAS-2B cell exposed to 2.4 μg/mL MWCNT-7 for 24 h. The nuclear envelope is indicated by the black arrows. Some of the MWCNT that are enclosed in the cell cytoplasm are indicated by green arrows. Several of the indicated MWCNT at the top of the micrograph (larger green arrows) appear to be within membrane bound vesicles while other MWCNTs within the cell cytoplasm (small green arrows) at the bottom of the micrograph are not membrane bound. The single red arrow indicates a MWCNT within the nucleus. The MWCNT within the nucleus is not bound by a lipid membrane. Magnification bar is 2.5 μm. (TIF 948 kb) [file 12989_2019_318_MOESM1_ESM.tif]

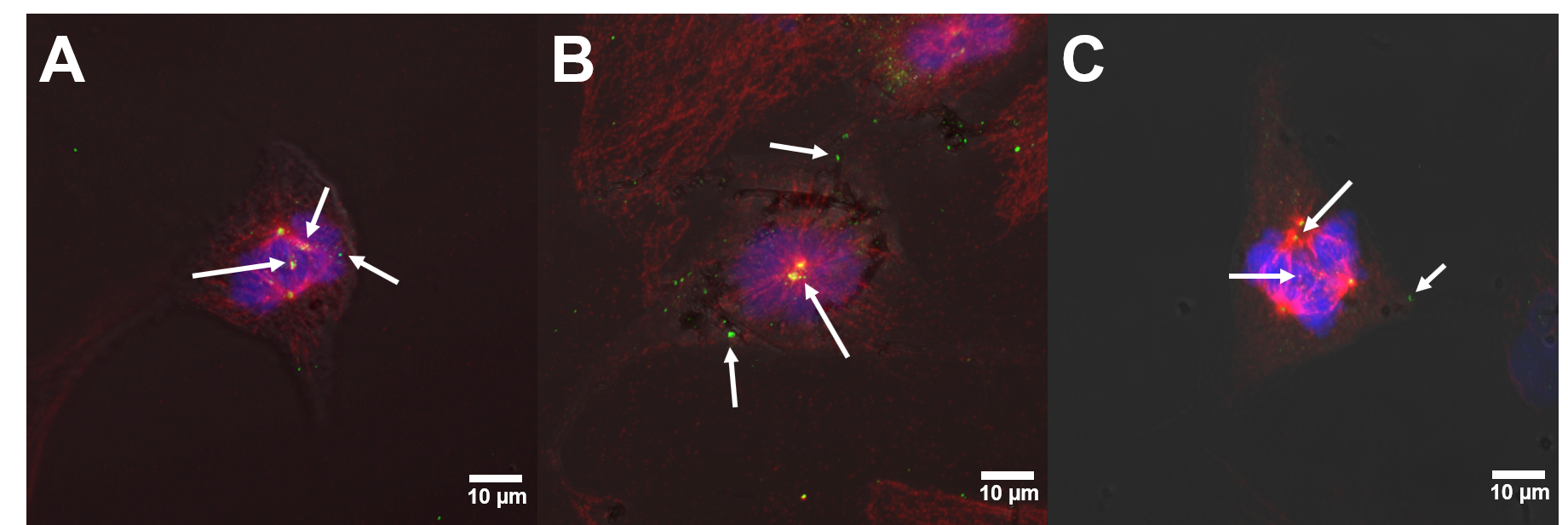

Supplement: Supplementary file 2 — Figure S2. Fragmented centrosomes cluster into one pole in BEAS-2B cells exposed to each MWCNT material. A-C) DNA blue, centrosomes are green, and mitotic spindle is red. A) MWCNT-HT, bipolar spindle. B) MWCNT-7, monopolar spindle. C) MWCNT-ND, multipolar spindle. White arrows point to clusters of fragmented centrosomes. Magnification bar is 10 μm. (TIF 1192 kb) [file 12989_2019_318_MOESM2_ESM.tif]

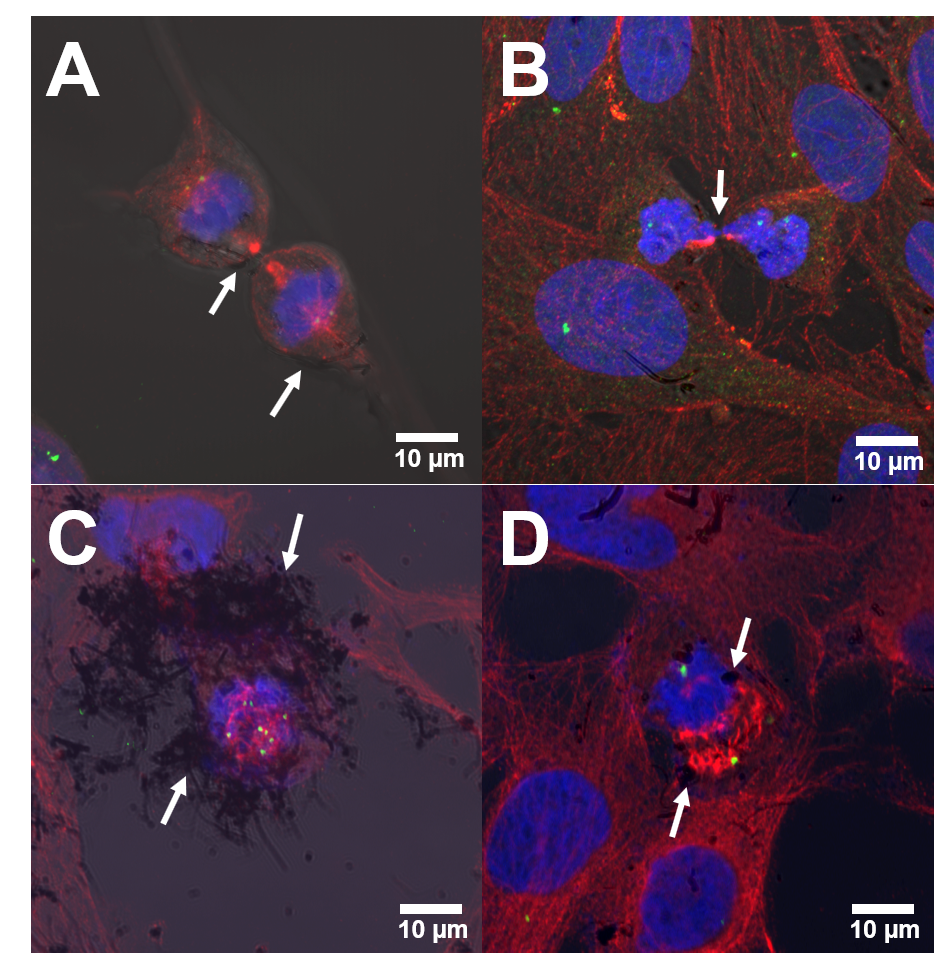

Supplement: Supplementary file 3 — Figure S3. MWCNT can interfere with spindle attachment to the centromere to produce supernumerary centrosomes, misaligned DNA, and centrosome fragmentation that can be so great a normal mitotic spindle cannot be formed in BEAS-2B cells exposed to MWCNT material for 24 h. A-D) DNA blue, centrosomes are green, and mitotic spindle is red. A) MWCNT-ND; supernumerary centrosomes. B) MWCNT-HT, C & D) MWCNT-7; misaligned DNA and catastrophic spindle morphology. White arrows point to MWCNT material within the bridge of cytokinesis (A & B) or MWCNT interacting with the DNA, centrosomes, and mitotic spindle (C & D). Magnification bar is 10 μm. (TIF 1533 kb) [file 12989_2019_318_MOESM3_ESM.tif]
